# Endoscopic ultrasound-guided ethanol injection with prophylactic pancreatic stenting for a pancreatic neuroendocrine neoplasm

**DOI:** 10.1055/a-2603-5560

**Published:** 2025-06-03

**Authors:** Kazuyuki Matsumoto, Yuki Fujii, Daisuke Uchida, Akihiro Matsumi, Kazuya Miyamoto, Ryosuke Sato, Motoyuki Otsuka

**Affiliations:** 1 92057 Department of Gastroenterology and Hepatology, Okayama University Hospital, Okayama, Japan

Endoscopic ultrasound (EUS)-guided ablation has been proposed as a viable treatment option for patients with small pancreatic neuroendocrine neoplasms (pNENs) 1 2 3 4 5 . However, ablation therapy is limited by the increased risk of pancreatic duct injury when treating lesions located near the main pancreatic duct (MPD). Prophylactic endoscopic pancreatic duct stenting (EPS) is considered effective in reducing the risk but no details of these cases have been described. We present a case of a small pNEN located near the MPD that was successfully treated with EUS-guided ethanol injection (EUS-EI) following prophylactic EPS, with complete ablation achieved without complications.

A pancreatic tumor was detected in a 59-year-old woman by abdominal ultrasound at an annual health check-up. Contrast-enhanced computed tomography showed a 15-mm hypervascular tumor in the pancreatic head ( Fig. 1 **a** ). The tumor was pathologically diagnosed by EUS-guided fine-needle biopsy as pNEN, grade 1. The patient was referred to our hospital for treatment with EUS-EI as a low-invasive treatment; however, EUS showed the tumor was located only 1 mm from the MPD ( Fig. 1 **b** ). Considering the risk of pancreatic duct injury due to ethanol treatment, prophylactic EPS (Geenen, 5 Fr, 12 cm; Cook Medical, Bloomington, Indiana, USA) was performed one month before the procedure ( Fig. 2 ). We started injecting ethanol into the part of the tumor closest to the MPD, and a total of 1.5 mL of ethanol was injected into three tumor locations until hyperechoic bubbles reached the tumor margin ( Video 1 ). The patient was discharged three days later. At one month after the procedure, the tumor was completely ablated with no complications ( Fig. 3 **a** ) and the pancreatic stent was removed. At the six-month follow-up, the tumor had completely disappeared and there were no complications such as MPD obstruction ( Fig. 3 **b** ). The patient has been followed up for two years, with no recurrence or other issues.

**Fig. 1:**
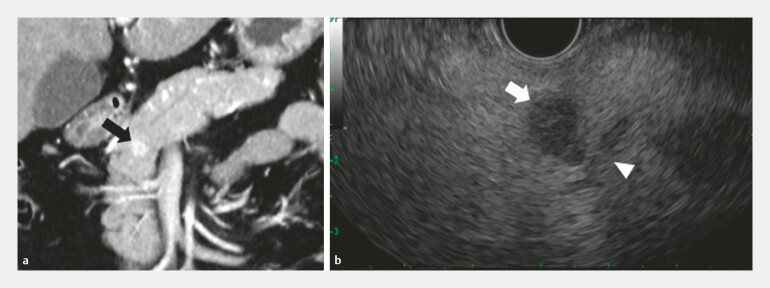
**a** Contrast-enhanced computed tomography (CE-CT) shows a hypervascular tumor 15 mm in diameter in the pancreatic head (arrow). **b** The tumor is visualized on endoscopic ultrasound as a hypoechoic lesion (arrow) located 1 mm from the main pancreatic duct (arrowhead).

**Fig. 2:**
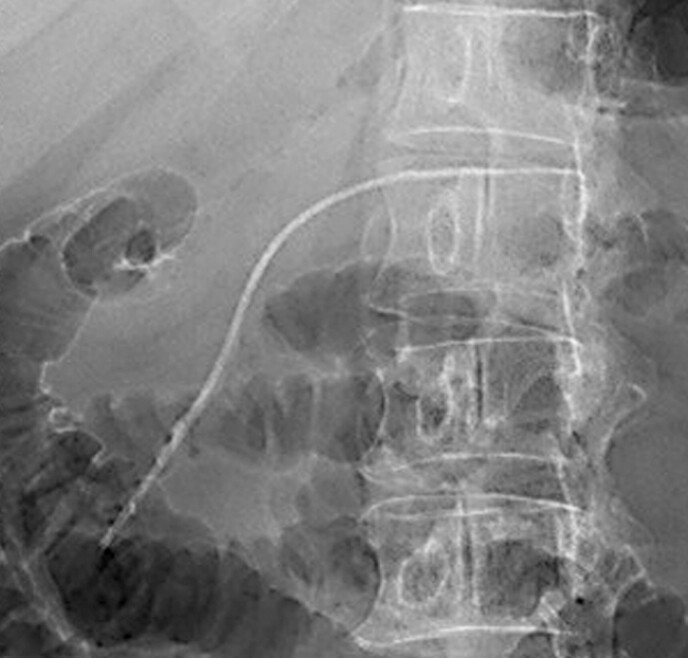
Endoscopic retrograde cholangiopancreatography image showing placement of the prophylactic pancreatic stent (5 Fr, 12 cm).

**Fig. 3:**
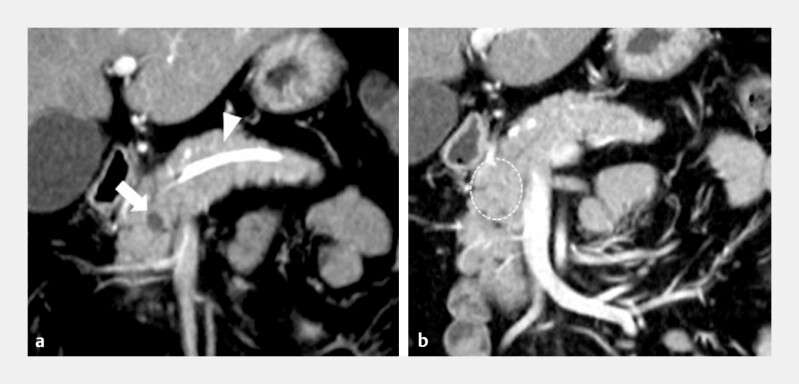
**a** CE-CT findings one month after the procedure. There is no enhanced area at the periphery of the tumor (arrow) and the pancreatic duct stent is retained (arrowhead). **b** CE-CT findings six months after the procedure. The previously enhancing areas of the tumor could no longer be detected on CE-CT (dotted circle) and there were no complications.

Successful endoscopic ultrasound-guided ethanol injection for a small pancreatic neuroendocrine tumor with prophylactic endoscopic pancreatic stenting.Video 1

Endoscopy_UCTN_Code_TTT_1AS_2AI

## Notes

Endoscopy E-Videos https://eref.thieme.de/e-videos
*E-Videos* is an open access online section of the journal *Endoscopy* , reporting on interesting cases and new techniques in gastroenterological endoscopy. All papers include a high-quality video and are published with a Creative Commons CC-BY license. Endoscopy E-Videos qualify for HINARI discounts and waivers and eligibility is automatically checked during the submission process. We grant 100% waivers to articles whose corresponding authors are based in Group A countries and 50% waivers to those who are based in Group B countries as classified by Research4Life (see: https://www.research4life.org/access/eligibility/ ). This section has its own submission website at https://mc.manuscriptcentral.com/e-videos .

## References

[1] Matsumoto K Kato H Itoi T Efficacy and safety of endoscopic ultrasonography-guided ethanol injections of small pancreatic neuroendocrine neoplasms: a prospective multicenter study. Epub ahead of print Endoscopy 2025 57 321 329 10.1055/a-2452-4607. 39454635 PMC11997695

[2] So H Ko SW Shin SH Comparison of EUS-guided ablation and surgical resection for nonfunctioning small pancreatic neuroendocrine tumors: a propensity score-matching study Gastrointest Endosc 2023 97 741 751 10.1016/j.gie.2022.11.004 36400239

[3] Crinò SF Napoleon B Facciorusso A Endoscopic ultrasound-guided radiofrequency ablation versus surgical resection for treatment of pancreatic insulinoma Clin Gastroenterol Hepatol 2023 21 2834 284300 10.1016/j.cgh.2023.02.022 36871765

[4] Imperatore N de Nucci G Mandelli ED Endoscopic ultrasound-guided radiofrequency ablation of pancreatic neuroendocrine tumors: a systematic review of the literature Endosc Int Open 2020 8 E1759 E1764 10.1055/a-1261-9605 33269308 PMC7671767

[5] Matsumoto K Kato H Kawano S Efficacy and safety of scheduled early endoscopic ultrasonography-guided ethanol reinjection for patients with pancreatic neuroendocrine tumors: Prospective pilot study Dig Endosc 2020 32 425 430 31580507 10.1111/den.13552

